# Thermo-Oxidative Destruction and Biodegradation of Nanomaterials from Composites of Poly(3-hydroxybutyrate) and Chitosan

**DOI:** 10.3390/polym13203528

**Published:** 2021-10-14

**Authors:** Anatoly A. Olkhov, Elena E. Mastalygina, Vasily A. Ovchinnikov, Tatiana V. Monakhova, Alexandre A. Vetcher, Alexey L. Iordanskii

**Affiliations:** 1 Scientific Laboratory “Advanced Composite Materials and Technologies”, Plekhanov Russian University of Economics, 36 Stremyanny Ln, 117997 Moscow, Russia; aolkhov72@yandex.ru (A.A.O.); elena.mastalygina@gmail.com (E.E.M.); fizhim@rambler.ru (V.A.O.); 2 N.M. Emanuel Institute of Biochemical Physics, Russian Academy of Sciences, 4 Kosygin St., 119991 Moscow, Russia; tvmonakhova@ya.ru; 3 N.N. Semenov Federal Research Center for Chemical Physics, Russian Academy of Sciences, 4 Kosygin St. 4, 119334 Moscow, Russia; aljordan08@gmail.com; 4 Institute of Biochemical Technology and Nanotechnology (IBTN), Peoples’ Friendship University of Russia (RUDN), 6 Miklukho-Maklaya St., 117198 Moscow, Russia; 5 Complementary and Integrative Health Clinic of Dr. Shishonin, 5 Yasnogorskaya St., 117588 Moscow, Russia

**Keywords:** poly(3-hydroxybutyrate), chitosan, electrospinning, thermal oxidation, biodegradation, Sturm’s method

## Abstract

A complex of structure-sensitive methods of morphology analysis was applied to study film materials obtained from blends of poly(3-hydroxybutyrate) (PHB) and chitosan (CHT) by pouring from a solution, and nonwoven fibrous materials obtained by the method of electrospinning (ES). It was found that with the addition of CHT to PHB, a heterophase system with a nonequilibrium stressed structure at the interface was formed. This system, if undergone accelerated oxidation and hydrolysis, contributed to the intensification of the growth of microorganisms. On the other hand, the antimicrobial properties of CHT led to inhibition of the biodegradation process. Nonwoven nanofiber materials, since having a large specific surface area of contact with an aggressive agent, demonstrated an increased ability to be thermo-oxidative and for biological degradation in comparison with film materials.

## 1. Introduction

Nowadays, much attention in world science is devoted to the creation of a new class of functional biodegradable highly porous materials based on ultrathin and nanoscale fibrous fibrillar structures with a wide variety of specific characteristics: physical, mechanical, sorption, and diffusion properties [1,2,3]. Such materials, based on synthetic and biopolymers, are widely used in biology, medicine, cell engineering, separation and filtration processes, reinforced composites, electronics, analytics, sensor diagnostics, as eco-sorbents for cleaning the environment from emergency spills of oil products and heavy metal compounds, and in many other innovative applications [4,5,6].

With the employment of traditional technologies, such as extrusion of polymer melts and solutions, spinneret drawing, etc., technological difficulties arise in obtaining fibers with a diameter of less than 1 µm [7]. Namely for this reason, currently, an electrospinning (ES) method, as a versatile and relatively simple method for forming ultra-thin and nanofibers with a diameter in the range from 10 nm to 10 µm from polymer solutions and melts, is employed [8]. The process is based on pulling a drop of solution or melt, formed at the end of a capillary, by the action of electrostatic forces applied to the solution or polymer melt, resulting in the formation of an ultra-thin nonwoven fiber [9,10].

Earlier, on the example of PHB and a number of its compositions with other polymers [11,12,13], the physicochemical, dynamic, and transport characteristics of film biodegradable matrices, microparticles, and microcapsules of PHB were studied for prolonged and targeted drug delivery [14,15]. High biocompatibility, controlled biodegradation into environmentally friendly products (carbon dioxide and water), and satisfactory mechanical characteristics make it possible to consider this polymer for the creation of innovative disposable products in food, packaging, medical, and environmental areas [11,16].

For the effective regulation of operational properties, combined (composite or mixed) materials based on PHB and other biopolymers, various organic and inorganic dispersed and fibrous fillers are currently available. Such composites, due to their heterophase and high heterogeneity, create, for example, a multiform dynamic of transport of low-molecular substances, due to the cooperation of the processes of structure formation (crystallization, plasticization, and swelling) and the kinetics of biodegradation of one of the components [17,18].

The most promising system from this view is a blend of PHB and CHT [19]. As was shown in the example of film matrices [20], the combination of PHB and CHT allowed to create of a new generation of amphiphilic fully biodegradable composite systems with an increased sorption capacity, a controlled rate of biodegradation, and the ability to implement various kinetic profiles of prolonged release of a low-molecular-weight substance in a wide time range (from weeks to months).

The structural organization and resistance to aggressive media of ultra-fibrous materials based on PHB and ultra-low concentrations of CHT (0.05–0.3 wt%) have been already analyzed [21]. The effect of CHT on both the crystalline and amorphous phases was shown: the addition of 0.1–0.2 wt% of CHT to the PHB structure increases the enthalpy of melting of PHB crystallites by almost 20%, which, in our opinion, is associated with the nucleating effect of chitosan, while the proportion of dense amorphous regions of PHB increases. The oxidation of PHB-CHT fibrous matrices with an ozone-oxygen mixture for up to four hours contributes to the compaction of amorphous regions, restricting molecular mobility, due to the predominant process of crosslinking of molecules over their oxidative destruction.

Hence, we would like to study in detail the structural organization and processes of thermo-oxidative and biodegradation of composite film and nonwoven fiber materials based on PHB with a high (40–70 wt%) content of CHT.

## 2. Materials and Methods

PHB (16F series) was obtained by microbiological synthesis by Biomer (Schwalbach am Taunus, Germany) with a viscosity-average molecular mass (M_ν_) of 4.6 × 10^5^ g·mol^−1^, with a degree of crystallinity around 59%, and CHT in the form of a fine powder with an average particle size of 1 ÷ 5 µm (Bioprogress CJSC, Shchyolkovo, Russian Federation) with the M_ν_ of 4.4 × 10^5^ g·mol^–1^ and the degree of deacetylation of 82.3% were employed.

According to the available publications, PHB is highly soluble in chloroform, dichloroethane, dimethylformamide, pyridine, dioxane, 1 M NaOH solution, higher alcohols, camphor. The more accessible and less toxic of these solvents are chloroform and dimethylformamide (DMF). The calculation of the compatibility of these solvents with PHB was carried out on the basis of a comparison of the solubility parameters by three different methods (according to Small, according to Hoya, and according to Van Krevelen). The average solubility parameter for PHB was 9.23, for chloroform 10.2, for DMF 12.5. Based on the data obtained, the solvent chloroform was selected as the most suitable in terms of solubility and toxicity.

To obtain the fibers, forming solutions of PHB in chloroform were prepared. The concentration of PHB in the solution was 7 wt%. The content of CHT in the forming solution was 40, 50, and 60 wt%, relative to the mass of PHB. Forming solutions of PHB with CHT were prepared at a temperature of 60 °C using an automatic high-speed agitator and an ultrasonic bath. The fibers were obtained by ES using a single-capillary laboratory setup with a capillary diameter of 0.1 mm, voltage of 12 kV, distance between the electrodes of 18 cm, and electrical conductivity of 10 µScm^−1^ [5,22].

In a comparison, samples of PHB/CHT were prepared in the form of films in several steps. First, PHB film was compressed from granules on the glass surface at a temperature of (180 ± 5) °C and a pressure of 10^7^ Pa. The resulting film was crushed and dissolved in chloroform. CHT powder was added to the solution with vigorous stirring using a high-speed mechanical stirrer at 1000 rpm, Disperser IKA T18 digital ULTRA TURRAX (IKA-Werke GmbH & Co. KG, Staufen, Germany), until a homogeneous suspension was obtained. Then, the suspension was subjected to exposure by simultaneous action of stirring at 500 rpm, 50 °C, 40 kHz for 30 min on an overhead stirrer LABTEH OS-20LT (Labteh Ltd., Moscow, Russian Federation) in ultrasonic bath Vilitek VBS-10DS (Vilitek Ltd., Moscow, Russian Federation). The resulting suspension was poured into Petri dishes, covered with a lid, and conditioned at room temperature until the films were completely dry.

The morphology of fibrous materials was studied by scanning electron microscopy (SEM) on Hitachi TM-1000 scanning electron microscope (Hitachi, Ltd., Tokyo, Japan). Changes in the microstructure of the samples before, during, and after the biological degradation was recorded with an Axio Imager Z2m optical microscope (Carl Zeiss, Jena, Germany) in transmitted (TL) and reflected (RL) light at magnifications of 50, 200, and 500×.

The study of thermophysical characteristics (temperature and enthalpy of melting) was carried out with a differential scanning calorimeter (DSC) DSC 214 Polyma (NETZSCH-Geratebau GmBH, Selb, Germany) according to ISO 11357-3:2018. The heating of the samples was carried out in the temperature range of 25–180 °C at a scanning speed of 10 °C/min. The weight of the sample was 10 ± 0.1 mg. The temperature scale and enthalpy of melting were calibrated against an indium standard sample.

The analysis of the chemical composition was carried out according to the manufacturer’s recommendation on the Bruker Lumos IR Fourier microscope (Bruker Corp., Bremen, Germany) at the temperature of (24 ± 2) °C in the range of wave numbers 400–4000 cm^−1^. The analysis was carried out by attenuated total reflection (ATR) using a diamond crystal.

The study of biological degradation was carried out under the action of soil microorganisms for the release of CO_2_ (Sturm method) according to ISO 14855-1:2012. To obtain an inoculum, compost was prepared according to Russian Standard GOST 9.060-75: equal proportions of sand, garden soil, and horse manure were mixed and conditioned for 60 days. Then, 860 g of prepared compost was mixed with 2 L of double-distilled water and filtered through a sieve with a mesh size of 0.5 mm. Subsequently, to purify the inoculum, the liquid part was subjected to separation in a laboratory centrifuge Hettich EBA 200 (Andreas Hettich GmbH & Co. KG, Tuttlingen, Germany) for 5 min at 1500 rpm, after which the liquid fraction was taken. Thereafter, the resulting suspension was passed through filter paper three times. The resulting soil concentrate was diluted with double distilled water to a volume of 9 L. The volume of round-bottomed test flasks was 500 mL. Six test flasks with inoculum without samples were bubbled with CO_2_-free air for 72 h before the start of the biodegradation experiment. The viability of bacteria in the soil concentrate (inoculum) was analyzed using a Polar 3 ToupCam 5.1 MP optical microscope (Micromed, Shenzhen, China) at 400 × magnification. In the process of biodegradation of materials by the microbiota of the inoculum, carbon dioxide was released. A 0.0125 M Ba (OH)_2_ solution was used as an absorbent. The amount of evolved carbon dioxide was estimated by titration with 0.05 M HCl solution and was calculated by the formula: CO_2_ (mg) = 1.1 × V(HCl), where V(HCl) is the amount of hydrochloric acid used for titration (ml). For alkalimetric testing, a set for automatic titration Titrion (Econix-Expert, Ltd., Moscow, Russia) was used. With a known initial content of total organic carbon in the samples, the biodegradation index was calculated. The analysis of the accumulation of carbon dioxide was carried out with an interval of 2–4 days. The biodegradation test was carried out for 60 days, the number of test flasks was 6: flasks 1, 2, 3, and 4 containing samples of the test materials and 500 mL of inoculum, as well as flasks 4 and 5 containing only inoculum (zero control).

The kinetics of oxidation of the samples was investigated by determining the amount of absorbed oxygen by the manometric method using a special manometric device [23]. The analysis was carried out at an elevated temperature (140 ± 2) °C and an oxygen pressure of 500 torr on a manometric device with the absorption of volatile oxidation products with solid KOH.

## 3. Results

### 3.1. Morphology Analysis

At the first stage of our work, we studied the features of the morphology and kinetics of biodegradation of fibrous and film materials based on PHB/CHT blends. All film and fibrous samples in each group, regardless of the PHB/CHT ratio, showed approximately the same patterns of morphology formation. Therefore, to consider the comparative kinetics of biodegradation, we selected two samples of film and fibrous materials without CHT and with a CHT weight % of 40–50 (all over the text (X0/Y0) means PHB/CHT (*w*/*w*)%).

**Figure 1:**
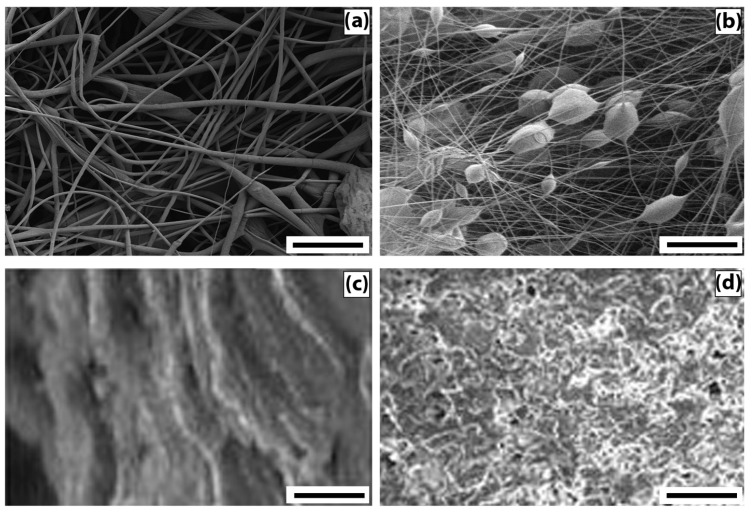
Images of the morphology of samples of nonwoven fibrous and film materials obtained by SEM: (**a**) PHB—fibers, (**b**) PHB/CHT—fibers (60/40), (**c**) PHB/CHT—film (50/50), and (**d**) PHB—film. The size of the scale bar—100 µm.

To describe the morphology of film composites and ultrathin fibers based on a mixed composition of PHB and CHT, micrographs of surfaces and edge cleavages were obtained, recorded by the SEM method (Figure 1).

The analysis of micrographs of surfaces and chips for mixed films demonstrates that as the content of PHB increases, the globularity of the structure becomes more and more pronounced. The globules look “inserted” into the CHT matrix, and, in general, both components form a heterophase structure. Note that, despite the heterophase nature, almost all ratios of polymer components retain the integrity of the PHB matrix with an increase in the concentration of CHT globules in it. It should be noted that the morphology of films with an equal polymer ratio is characterized by the separation of components with the formation of a two-layer structure. At a high content of CHT in the layer, an insignificant amount of PHB globules can be seen; however, in general, phase separation occurs quite noticeably. The formation of a bilayer structure should affect the diffusion, mechanical, and other physico-mechanical characteristics of the polymer composition.

The fibrous samples analysis reveals the morphology difference of the nonwoven materials from PHB and PHB/CHT. PHB fibers are randomly located, individual filaments of circular cross-sections with a diameter of 3.5 ± 2.5 μm. This diameter distribution is a consequence of the splitting of the primary jet during the ES of the polymer solution. The fibers contain single defects in the form of extended thickenings of arbitrary geometry. The origin of these defects is associated with the suboptimal electrical conductivity of the polymer solution [24]. PHB/CHT fibers, on the other hand, consist of cylindrical sections with an average diameter of 3 ± 1 μm and spherical thickenings slightly elongated along the fiber axis with an average size of 15 ± 5 μm. The presence of thickenings in the fiber structure is associated with particles of the dispersed phase of CHT. Dispersion of CHT in the form of relatively large particles is associated with the lack of solubility in CHCl_3_ during the preparation of ES solutions based on PHB. The solutions were instead suspensions.

### 3.2. Study of Biological Degradation

**Figure 2:**
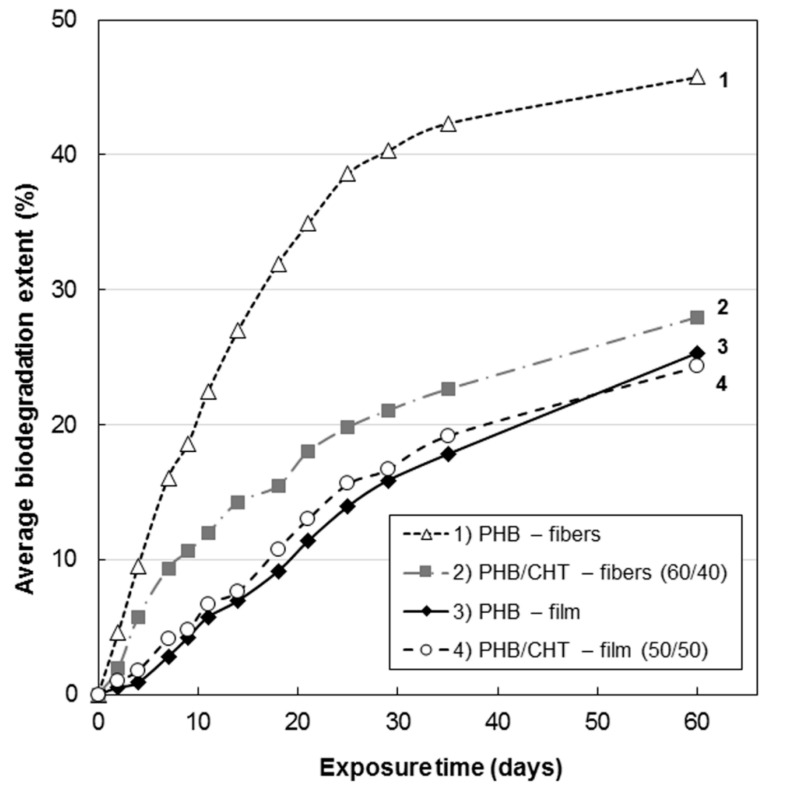
Kinetics of biodegradation (according to the Sturm method) of samples of nonwoven fibrous and film materials: (**1**) PHB—fibers; (**3**) PHB—film; (**2**) PHB/CHT—fibers (60/40); and (**4**) PHB/CHT—film (50/50).

The kinetics of biodegradation is demonstrated in Figure 2. In comparison with films, nonwoven materials were characterized by a higher rate of biodegradation, which is associated with a higher specific surface area and, therefore, increased accessibility for attack by microorganisms. Similar patterns have been described for nonwoven materials based on polylactic acid [25].

Analysis of the kinetic curves in Figure 2 suggests that the introduction of CHT into PHB leads to inhibition of the biodegradation process. This can be explained by the antibacterial properties of CHT, which are a consequence of the interaction of its positively charged amino groups with negatively charged phosphoryl groups of phospholipids of the bacterial cell wall, a violation of its integrity with changes in metabolism, which eventually leads to cell death [26].

In addition, CHT is capable of interacting with nucleic acids, thereby disrupting the biosynthesis of proteins and damaging the structural and functional complexes of the bacterial cell [27]. The fungicidal properties of CHT are explained by similar mechanisms.

Analysis of the nature of the kinetic dependences in Figure 2 allows distinguishing two characteristic areas in them. The first section up to 30 days is characterized by an intensive course of the process of hydrolysis of film and fibrous materials based on PHB. This feature is associated with the processes of degradation of the loose amorphous phase of PHB, intercrystalline regions, stressed regions at the PHB/CHT interface, and the CHT phase itself. The second section is characterized by a low rate of processes of hydrolytic degradation of materials, which we associate with the destruction of directly crystalline formations of polymers. It is known that crystalline grains have a high packing density of macromolecules and are resistant to aggressive environmental factors [28]. Their degradation is associated with low permeability for all types of low molecular weight liquids and gases [29].

**Figure 3:**
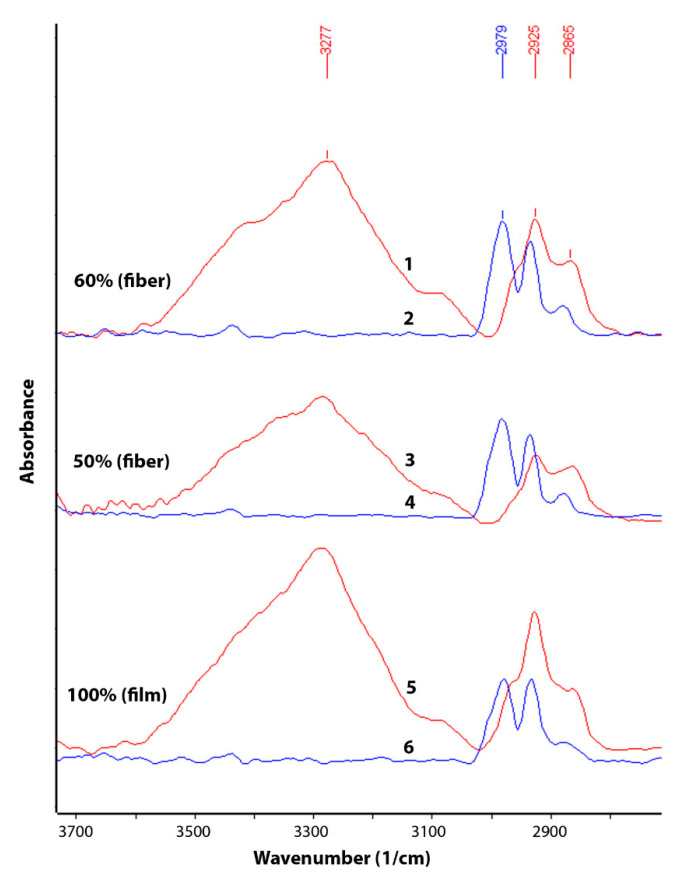
IR spectra (ATR method) in the wavelength range of 2700–3700 cm^−1^ of the initial samples (2,4,6) and samples after 60 days of testing by the Sturm method (1,3,5): PHB/CHT (fibers)—(60/40) (1 and 2), PHB/CHT (film)—(50/50) (3 and 4), PHB (film) (5 and 6).

**Figure 4:**
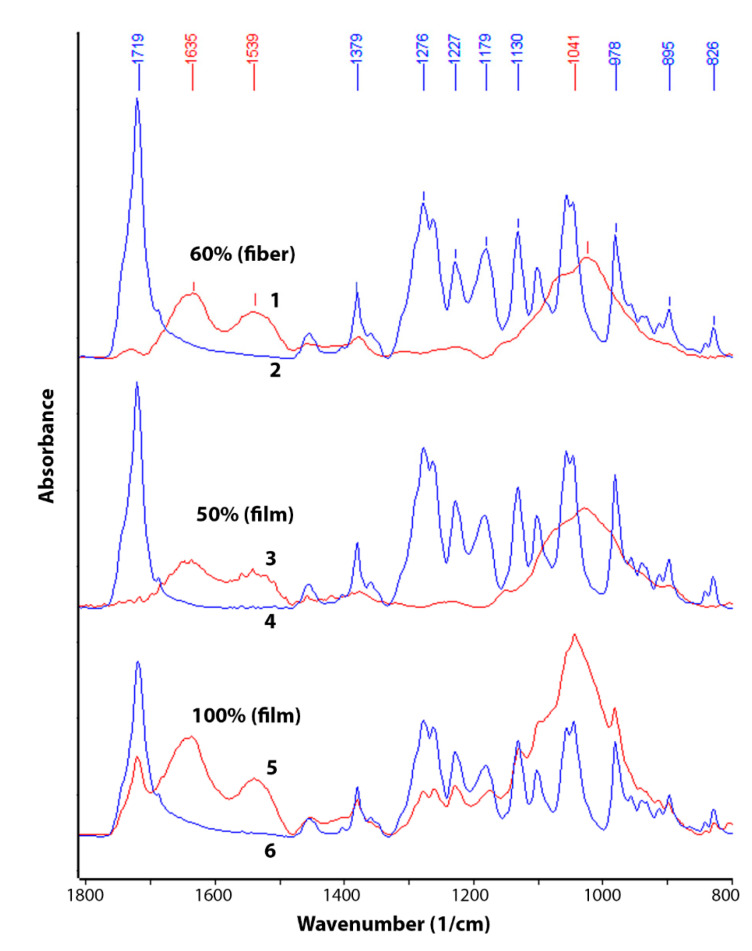
IR spectra (ATR method) in the wavelength range 800–1800 cm^−1^ of the initial samples (2, 4, 6) and samples after 60 days of testing by the Sturm method (1, 3, 5): PHB/CHT (fiber)—(60/40) (1 and 2), PHB/CHT (film)—(50/50) (3 and 4), PHB (film) (5 and 6).

The change in chemical composition during biodegradation was studied by FTIR (Figure 3 and Figure 4). An analysis was made of changes in the chemical composition of samples of materials PHB (film), PHB/CHT (film) (50/50), PHB/CHT (fibers) (60/40) after 60 days of tests for biodegradation under the influence of soil microflora. The PHB-based samples obtained by ES on a substrate (PHB—fibers) were severely damaged after testing, which made them difficult to analyze.

Since the analysis was carried out by the ATR method, changes in the outer layers of the material were monitored. It should be noted that the IR spectra of the initial samples of PHB (film) and PHB/CHT (film) (50/50) have similar absorption peaks in the entire wavelength range corresponding to pure PHB. For example, in the near-surface layers of the composite material with the equal content of components, PHB is predominantly located.

After 60 days of biodegradation in the soil inoculum, changes in the chemical composition were observed both in materials based on pure PHB and in composites based on PHB and CHT. Moreover, composite nonwoven materials were characterized by greater changes than film samples. When comparing the IR spectra of PHB (film) of the original and subjected to biodegradation, a significant increase in absorption bands was found in the regions of 3100–3600, 1580–1700, 1490–1580, and 950–1150 cm^−1^. In this case, the intensity of the absorption peak with a maximum at 1720 cm^−1^, characteristic of PHB and corresponding to stretching vibrations of carbonyl groups (C=O), significantly decreased [30].

The appearance of a diffuse absorption peak in the region of 3100–3600 cm^–1^ indicates the accumulation of hydroxyl groups (–OH) and amide groups (–NH_2_) in the near-surface layers of the material [31]. The emerging absorption peaks at 1530 and 1635 cm^–1^ correspond to bending vibrations of amide groups (–NH_2_) in primary amides [32]. There was also a significant increase in the absorption band at 1041 cm^−1^. This band corresponds to vibrations of C-O-C bonds and is characteristic of carbohydrates [33].

**Figure 5:**
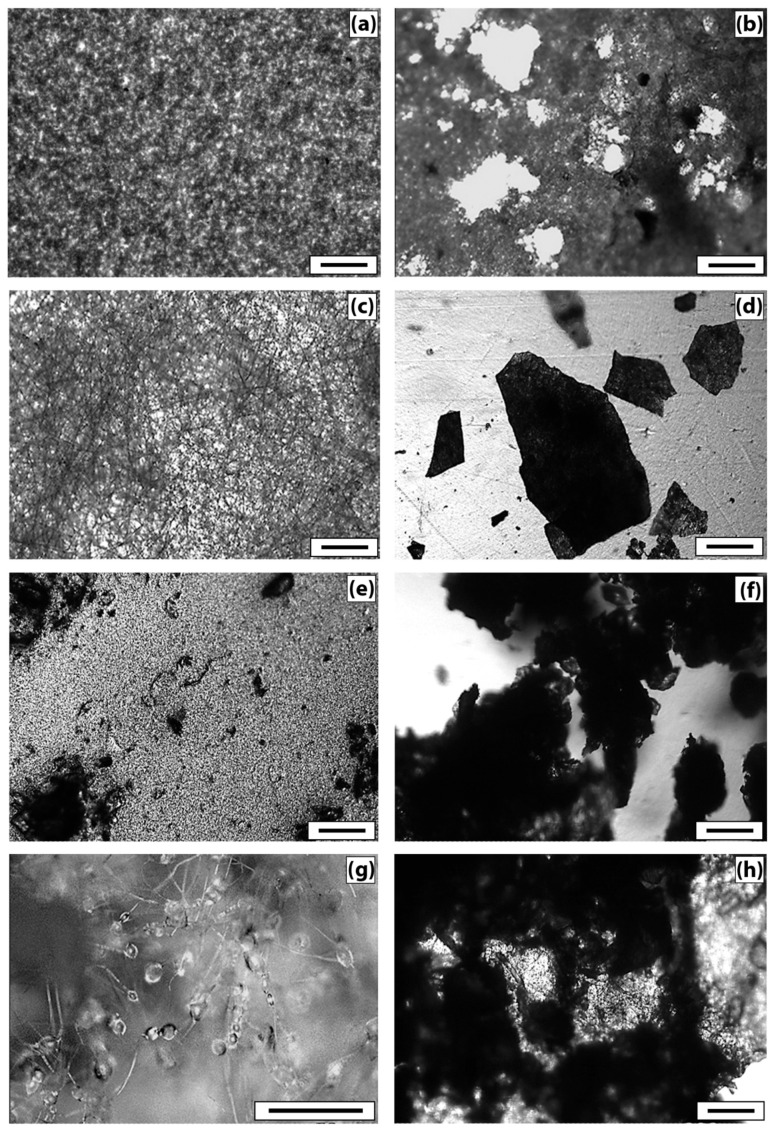
Optical micrographs of PHB (film) samples—(**a**) initial and (**b**) after 60 days of tests by the Sturm method; PHB (fiber)—(**c**) initial and (**d**) after 60 days of tests by the Sturm method; PHB/CHT (film) —(50/50)—(**e**) initial and (**f**) after 60 days of tests by the Sturm method; and PHB/CHT (fiber)—(60/40)—(**g**) initial and (**h**) after 60 days of tests by the Sturm method). The size of the scale bar—100 µm.

Changes in the microstructure of the samples were examined by optical microscopy. Figure 5 demonstrates that the PHB sample (film) (Figure 5a) has a fairly uniform structure, with some roughness on the surface. There are no violations of the film continuity. After tests by the Sturm method, violations of the integrity of the sample and numerous through-holes are visible (Figure 5b). The damage to the material is visible under the microscope–darkening of the material in the appearance of the micromycetes’ mycelium.

The original PHB sample (nonwoven) (Figure 5c) is characterized by a uniform fibrous structure. The average fiber diameter is 4.5 ± 1.5 µm. As a result of tests by the Sturm method, significant damage to the material is observed. The fibrous structure of the sample becomes almost indistinguishable, numerous fiber breaks and signs of microbiological damage are visible.

The original film sample of PHB/CHT (50/50) (Figure 5e) differs from the PHB film by a more heterogeneous structure. Granular areas of the structure and through defects are visible, which can adversely affect the physical and mechanical properties of the material. On the other hand, the loose structure provides access to the material for degradation agents. After biodegradation by the Sturm method, the number of defects increases, and the darkening of the material is observed.

The original nonwoven sample PHB/CHT (60/40) (Figure 5g) is characterized by an uneven fibrous structure, thickness differences, and spindle-shaped thickenings of 15–20 µm are visible. As a result of tests by the Sturm method, significant damage to the material is observed; however, the fibrous structure remains. Blind cavities and traces of microbiological damage appear.

### 3.3. Thermo-Oxidative Destruction

Under real conditions of biodegradation of products, the processes of hydrolysis and oxidation proceed simultaneously. In our experiment with the Sturm method, the samples were incubated in an aqueous inoculum, which led to the predominant contact of materials with water and a relatively small amount of oxygen dissolved in water. In this case, the processes of hydrolytic degradation of polymers predominantly took place. Therefore, in the second part of our study, we will separately consider the processes of oxidative degradation of PHB/CHT samples. For acceleration, the oxidation process was carried out at 140 °C in an oxygen atmosphere. This is the maximum possible temperature at which no noticeable changes in the chemical composition and supramolecular structure occurred in the samples.

**Figure 6:**
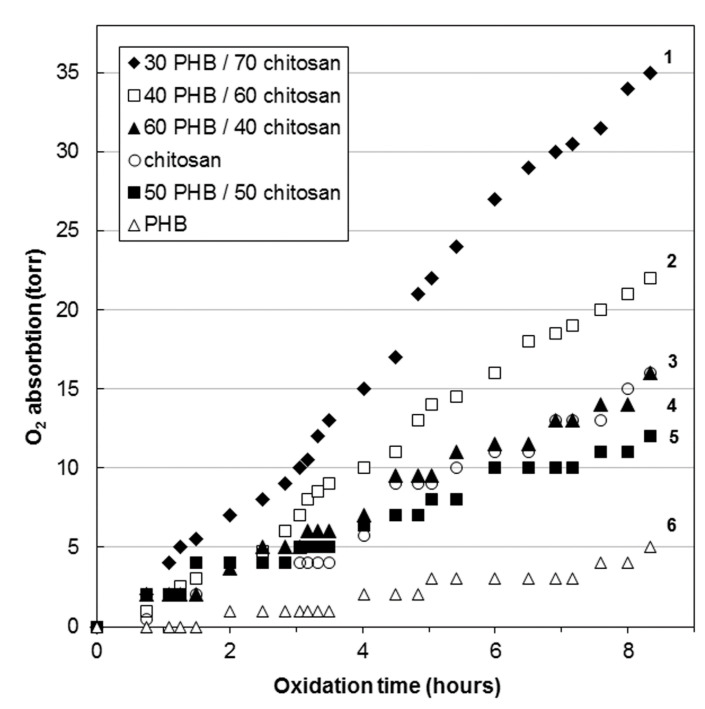
Kinetic curves of oxygen absorption by nonwoven fibrous materials PHB/CHT: (30/70)—1, (40/60)—2, (60/40)—3, (0/100)—4, (50/50)—5, and (100/0)—6.

Figure 6 demonstrates the kinetic dependences of the oxidation of PHB/CHT fibrous materials.

Figure 6 demonstrates the kinetic curve for PHB fibers has an induction period of about 90 min. This indicates the oxidation of the material in the diffusion mode. The sample oxidizes extremely slowly. The same effect was previously noted in the study of thermal oxidation of extrusion films based on blends of polyethylene/PHB [34], polyvinyl alcohol/PHB [35], and ethylene-propylene copolymer/PHB [36]. The increased resistance of PHB to oxidation is due to the high degree of crystallinity and the dense structure of the amorphous regions, which impede the diffusion of oxygen [37].

The kinetic curves of oxidation of mixed fibers containing 40–50 wt% of CHT are intermediate between pure PHB and CHT, but are relatively close to the kinetic curve of the latter. It should be noted that CHT is oxidized better than PHB. This is due to the better oxygen permeability. Such closeness of the oxidation curves of blends to CHT is explained by the fact that, in the range of CHT content of 40–50 wt%, phase inversion occurs in the mixed fibers and both components form a continuous network. It should also be noted that the analysis of the IR spectra (ATR) of blended fibers with a CHT content of 40–50 wt%, presented above, exhibited the presence of only the PHB phase in the surface layers. It is known that crystallization does not occur in the surface layers of films and fibers [38,39]. In this case, oxygen diffuses faster through the amorphous surface layer of PHB to the more reactive phase of CHT, and the oxidation of blends proceeds in a kinetic mode without an induction period typical for PHB fibers.

With an increase in the content of CHT in blends to 60–70 wt%, the structure of the fibers changes. The PHB phase becomes dispersed and acts as a binder for CHT particles during the formation of a nonwoven fibrous material. The kinetic curves of oxidation of these materials are significantly higher than of PHB and CHT themselves.

When heated, pure CHT is characterized by an endothermic peak at 50–100 °C, which is related to the process of moisture evaporation. In this case, CHT is thermostable up to 250 °C [40]. Therefore, CHT should not have a significant effect on the endothermic peak of PHB melting in the range of 160–170 °C. However, upon heating, a complex bimodal peak is observed on the thermograms of PHB/CHT blends. Therefore, when calculating the enthalpies of the effect for the initial materials, an apparent increase in the enthalpy of melting of PHB is observed. This is associated with the interaction of PHB and CHT molecules with the formation of hydrogen bonds, which are destroyed when the blends are heated, which contributes to the endothermic effect [41].

From the data presented in Table 1, at 500 min of thermal oxidation, the enthalpy drops by 30–40% in blends with a content of 60–70 wt% CHT and by 10% in blends with 40 wt% CHT. At the same time, the enthalpy of melting in PHB fibers increased by 30%. The increase in the enthalpy of melting is most likely associated with the processes of oxidative degradation of overstressed covalent bonds of the PHB main chain localized in the amorphous regions, which leads to additional crystallization. The decrease in the enthalpy of mixed samples can be explained by the processes of CHT oxidation, as a result of which the number of intermolecular hydrogen bonds with PHB macromolecules decreases.

At the same time, it should be noted that the change in the melting temperatures of the PHB phase in the mixed fibers changes insignificantly (3–4 °C), which indicates the invariability of the average crystallite size. According to Table 1, as the content of CHT increases, the fraction of the crystalline phase in PHB also increases, as evidenced by the value of the enthalpy of melting. Upon thermal oxidation for 500 min, the melting point of PHB crystallites practically does not change (3–4 °C), while the amount of the crystalline phase decreases significantly, especially in fibers containing 60–70 wt% of CHT.

## 4. Discussion

The interaction of PHB and CHT is contingent on forming hydrogen bonds. According to the available published data, the interaction of PHB and CT was proved through the formation of hydrogen bonds between the ester groups of PHB and the mobile protons of the amino and hydroxyl groups of CHT [42]. These interactions between polymers, in turn, affect the crystallization of PHB. The combination of the results of FTIR spectroscopy and the results of DSC, as well as SEM micrographs of the edge cleavages, also confirm that PHB and CHT interact with each other through the formation of hydrogen bonds between the ester groups of PHB and the amine groups of CHT.

The biodegradability of PHB-based materials is an important parameter. One of the objectives of this work was to study the kinetics of biodegradation of PHB/CHT materials under the influence of soil microorganisms and to reveal the effect of CHT on the biodegradation of PHB. It is well known that PHB is capable of hydrolysis [43,44], while enzymes secreted by microorganisms of the medium are capable of accelerating this process. In addition, an important factor for the biodegradation process is the availability of the material for attack by microorganisms, including its contact area with the environment.

There are several publications devoted to the degradation of PHB composites with other polymers in model media. As a rule, water or aqueous solutions of acids and alkalines are used as media. For example, the kinetics of degradation of fibrous scaffolds based on PHB with additions of CHT and bioglass were studied in the work [45]. The fibrous PHB/CHT (50/50) material was characterized by weight loss of 25% after conditioning in water at 37 °C for 60 days. The other researchers [46] demonstrated that PHB/CHT (85/15) blend experienced a weight loss of 10% after conditioning in water at 37 °C for 90 days. No studies are available on the biodegradability of PHB/CHT composites under the action of a combined microbiota that simulate the real conditions of materials degradation. 

In this work, the study of biological degradation was carried out under the action of soil microorganisms for the release of carbon dioxide. The amount of emitted carbon dioxide, measured using the Sturm method, is the most reliable criterion for the biodegradation of organic molecules. The results of this study showed that PHB-based fibrous material was characterized by the highest biodegradability (biodegradation rate was 45.8% after 60 days). Pure chitosan has slow biodegradation compared to PHB, about 12% for 30 days [47]. The addition of chitosan inhibited the biological degradation of PHB due to the antimicrobial properties of the former. Thus, for the fibrous material based on the blend PHB/CHT (60/40), the degree of biodegradation was 28.0% after 60 days of the experiment.

After the biodegradation process under the action of soil microbiota, significant changes in chemical composition and microstructure were observed. The emerging absorption peaks at 1530 and 1635 cm^–1^ attributed to amide groups and a significant increase in the absorption band at 1041 cm^−1^ (C-O-C bonds in carbohydrates) is likely related to the increased number of chitin molecules contained in fungal cell walls [48]. This fact once again confirms the biofouling of the samples by microorganisms with their subsequent destruction.

The complete disappearance of the peak at 1720 cm^−1^ for the blends PHB/CHT and a decrease in the intensity for the pure PHB after biodegradation test indicate the destruction of PHB followed by forming microdefects and integrity damage. According to optical microscopy analysis, the materials based on pure PHB were characterized by biodeterioration throughout the samples. The film and fibrous materials based on the blends PHB/CHT had numerous sites of biodeterioration.

The reports also revealed the effect of CHT on the ability of PHB to oxidative destruction. Some publications confirmed that during the mild oxidation of CHT, hydroxyl groups (C3 and C6 sites) are oxidized to carbonyl ones without the noticeable effect of the polymer backbone structure. The number of amino groups may also decrease [49]. However, CHT is not an oxidation catalyst. Usually, catalysts and photosensitizers are used to oxidize chitosan itself.

In this case, the supramolecular structure of PHB/CHT blends plays an important role in the process of oxygen sorption. As shown earlier [50], the amorphous phase of PHB fibers consists of dense and relatively “loose” regions. The presence of dense areas makes it difficult to diffuse low molecular weight substances and gases. However, when low-molecular substances are added to the forming solution, it leads to a change in the processes of structure formation of PHB, as a result of which an amorphous phase with an increased free volume is formed. The formation of a looser structure of the amorphous phase is a direct consequence of the inhibition of crystallization processes as a result of adsorption or electrostatic interaction with polar groups of low molecular weight substances. Because film and fibrous samples were formed from the solution, the increase of PHB crystallinity after the oxidative degradation could be explained by isothermal crystallization during the experiment. Thermal oxidation of PHB leads to the destruction of amorphous regions and an increase in the mobility of macromolecules, which facilitates the recrystallization of the most ordered PHB regions. An increase in free volume in composite fibers converts the diffusion mode of oxidation into a kinetic one. Accelerated oxidation of blended fibers with a CHT content of more than 60 wt% can also be a consequence of the presence of intermolecular interaction between PHB and CHT, which have polar groups in the main chain (oxygen-containing—PHB, amine—CHT). In this case, the resulting physical bonds with CHT particles prevent the convergence of macromolecule regions in the amorphous phase of PHB, creating an additional free volume. Due to intermolecular interaction at the phase boundary of PHB/CHT, a layer more reactive to oxidation is formed, characterized by a stressed state of a portion of macromolecules [51]. This occurs during the ES. When a fiber is pulled in the field of action of an electrostatic force in the PHB phase, stresses arise at the interface with CHT due to the inability of the latter to perform deformation stretching.

## 5. Conclusions

This work analyzes the patterns of structure formation of film and fibrous biocomposites based on blends of natural polymers—poly(3-hydroxybutyrate) and chitosan. Chitosan having antimicrobial activity due to its cationic residue is an efficient additive for biomedical materials based on fully biodegradable PHB. Since chitosan possesses hydroxy and amino groups, PHB can be chemically bonded with it by blending. The use of a complex of such structure-sensitive methods like FTIR spectroscopy, DSC, and SEM made it possible to establish that PHB and CHT interact with each other due to the presence of polar reactive ester (PHB) and amino groups (CHT). This affects the formation of the supramolecular structure of materials, which, in turn, becomes more or less reactive with oxygen or water in the course of natural degradation.

The peculiarities of the composition and structure of composites had a significant effect on the oxidative destruction and biological degradation of fibrous and film materials based on blends of PHB and CHT. The lack of compatibility of the polymer components in the system is not a disadvantage. The availability of immiscible heterogeneous structures is generally higher than that of homogeneous continuous structures. Since heterogeneous structural elements are more actively attacked by water and other hydrolyzing agents (acids, enzymes), then, by regulating the degree of heterogeneity and sizes of structural elements, there is a potential opportunity to control the rate of hydrolytic decomposition of the polymer system.

The obtained results show a high biodegradability of the materials based on PHB and CHT and allow predicting the complete degradation of the materials within seven months under biocompost conditions (for ES blend material PHB/CHT (60/40)). At the same time, elevated temperatures will accelerate the degradation of materials due to the occurrence of oxidative destruction. It was found that the introduction of high concentrations (over 60 wt%) of CHT into film and fibrous materials based on PHB significantly accelerates the processes of oxidative and hydrolytic destruction, but leads to a slight inhibition of the growth of micromycetes at the initial stage.

In addition, the effect of the type of material (film or ES fibrous material) on the rate of biodegradation under the influence of soil microbiota was shown, which is due to the different accessibility for attack by microorganisms.

Thus, the developed materials on the PHB/CHT basis have an increased ability for biodegradation and oxidative degradation, as well as have potential antibacterial properties due to the presence of CHT. These composite materials can be recommended for the creation of disposable biodegradable sorbents, medicine materials, and hygiene products.

## Figures and Tables

**Table 1:** Changes in thermophysical parameters melting temperature (T_m_) and enthalpy (ΔH_m_) of PHB/CHT fibrous materials during oxidation for 500 min at 140 °C.

(*w*/*w*) Ratio of PHB/CHT Samples	Initial		Oxidazed	
	T_m_, °C (δ ± 1°C)	Δ H_m_, J·g^−1^ (δ ± 5 J·g^−1^)	T_m_, °C (δ ± 1°C)	Δ H_m_, J·g^−1^ (δ ± 5 J·g^−1^)
30/70	169	106	160	79
40/60	163	125	166	72
60/40	162	69	159	63
100/0	166	65	162	87

## Data Availability

The data presented in this study are available on request from the corresponding author.
